# Blunted Behavioral and C Fos Responses to Acidic Fumes in the African Naked Mole-Rat

**DOI:** 10.1371/journal.pone.0045060

**Published:** 2012-09-17

**Authors:** Pamela Colleen LaVinka, Thomas J. Park

**Affiliations:** Department of Biological Sciences and the Laboratory of Integrative Neuroscience, University of Illinois at Chicago, Chicago, Illinois, United States of America; Barnard College, Columbia University, United States of America

## Abstract

Acidosis in the skin triggers activation of pain pathways and behaviors indicative of pain in vertebrates. The exception is the naked mole-rat, the only known vertebrate to show physiological and behavioral insensitivity to acid pain in the skin. The goal of the present study was to determine behavioral and physiological responses of this species to airborne acidic fumes, which would be expected to affect the trigeminal pain pathway in other species. Behaviorally, naked mole-rats did not avoid fumes from moderately high concentrations of acetic acid (10 and 20%), and c Fos labeling showed no increase in activity in the trigeminal nuclei and nucleus tractus solitarius. In contrast, these concentrations triggered behavioral aversion and increased Fos activity in other laboratory rodents. For a very high concentration of acetic acid (50%), naked mole-rats showed significant avoidance behavior and increased Fos labeling in the nucleus tractus solitarius caudal region, which receives vagal chemosensory information. However, there was no increase in trigeminal labeling, and in fact, activity significantly *decreased*. This pattern is opposite of that associated with another irritant, ammonia fumes, which elicited an increase in trigeminal but not nucleus tractus solitarius Fos labeling, and no behavioral avoidance. Behavioral avoidance of acidic fumes, but no increased labeling in the trigeminal pain nucleus is consistent with the notion of adaptations to blunt acid pain, which would be advantageous for naked mole-rats as they normally live under chronically high levels of acidosis-inducing CO_2_.

## Introduction

Acidification of the nasal epithelium from inhaling acidic fumes or high concentrations of CO_2_ evokes pain in humans and other mammals [1], [2]. The goal of the present study was to determine the sensitivity of African naked mole-rats to acidic fumes. This is of interest in naked mole-rats for the following reasons. Previous experiments with this species showed that it is the only known vertebrate whose skin is naturally insensitive to acid [3]. These animals did not respond behaviorally to application of acidic saline in the skin, nor did their sensory nerve fibers show physiological responses to acidic saline. Recently, Smith, et al. [4] described the molecular mechanism for the naked mole-rat’s insensitivity to acid. The voltage-sensitive sodium channel Nav1.7 in this species has a gene variant that prevents acid-induced action potential initiation, even though acid sensors (e.g. TRPV1 and ASIC3) are functional.

Our working hypothesis about a selective pressure for acid insensitivity in this species is an adaptation for living under a chronically high CO_2_ environment [5]. Naked mole-rats live in large, very social groups [6], [7] in underground burrows with poor air circulation, resulting in high levels of CO_2_ (and low levels of oxygen). Elevated carbon dioxide drives tissue acidosis and pain [8], [9]. In the mammalian nasal cavity, trigeminal nerve fibers that respond to CO_2_/acidosis are primarily small caliber, unmyelinated C fiber nociceptors [10], [11]. The nociceptor ion channel TRPA1 is activated by CO_2_-derived acidosis as well as weak acids such as acetic acid, and it is gated by intracellular protons that cross the cell membrane during exposure [2], [9].

Stimulation of TRPA1 receptors in the nasal epithelium leads to activation of afferents in the trigeminal nerve [2], [9], and post-synaptic cells in the spinal trigeminal nucleus which can be readily detected using c Fos immunolabel [12]–[15].

When activated, the trigeminal chemosensory system triggers a number of physiological and behavioral responses that function to protect the animal. Physiological responses include mucus secretion and modulation of respiration rate and heart rate [16]. Behaviors include responses indicative of pain such as rubbing the nose, and avoidance responses such as withdrawing from or avoiding the stimulus [17]–[19].

Our present results show that naked mole-rats avoid acidic fumes, but their threshold for behavioral avoidance is much higher than the thresholds of laboratory rats and mice and another African mole-rat species, Damaraland mole-rats (*Fukomys damarensis)*. C Fos labeling showed that the avoidance behavior in naked mole-rats was not driven by increased activity of the trigeminal pain pathway. However, increased labeling in the nucleus tractus solitarius (NTS) suggests that this pathway may be involved in the naked mole-rat’s avoidance of acidic fumes.

## Methods

### Ethics Statement

All animal protocols were approved by the University of Illinois at Chicago Institutional Animal Care and Use Committee.

### Animals

Experiments were conducted on naked mole-rats (*Heterocephalus glaber*), Damaraland mole-rats (*Fukomys damarensis*), Sprague-Dawley and Long-Evans rats, and C57BL/6 mice. The naked mole-rats were non-breeding animals between 20 and 40 grams in weight and at least one year in age. The naked mole-rats were housed under semi-natural conditions in an artificial burrow system within a colony room. Because naked mole-rats are poikilotherms, the room where they were housed in was maintained at 27.8°C and 45–65% relative humidity [20]. Rats, mice, and Damaraland mole-rats (which are also African mole-rats but not poikilothermic) were housed in static rodent cages, under conventional laboratory vivarium conditions (23°C and 20–35% relative humidity).

### Behavioral Procedures

#### Avoidance of air borne irritants

Animals were placed in a four-arm arena (often referred to as a plus maze) made from black plexiglass, with a sponge affixed to the end of each arm. Each open arm was 55 cm in length, 15 cm in height, and 10 cm in width. In one test, one of the sponges was saturated with acetic acid, and the remaining three sponges were saturated with water. Animals were tested in the rooms in which they were housed. Animals were placed into the arena one at a time at the center, and allowed to explore freely for 20 minutes which they readily did. During this time, we recorded the cumulative amount of time spent within 10 cm of each sponge. The idea was that if an animal perceived acetic acid fumes as aversive, then it would spend less time near the sponge saturated with acid solution compared to the other sponges. To control for possible spatial cues, after each individual animal was tested, the arena was wiped clean with ethanol, rotated 90 degrees, and the sponges were re-positioned such that a given sponge (e.g. the acetic acid sponge) was not consistently in the same arm or the same spatial location relative to landmarks in the testing room.

We tested three concentrations of acetic acid solutions: 10, 20, and 50%. We also tested animals with 10% ammonia which is the concentration of household cleaning ammonia. Only time spent within ten centimeters of each saturated sponge, and therefore within the highest evaporative concentration of stimulant, was recorded. In a previous study, we reported results from testing naked mole-rats and laboratory rats with ammonia [12]. To complete the comparison across species and stimuli, we tested mice and Damaraland mole-rats with ammonia in the present study. Individual animals were only tested in one condition (i.e. all animals were naïve).

For statistical analysis, we first performed a 2-way ANOVA (VassarStats,Vassar College, Poughkeepsie, NY) to assess the main affects of species and aversive stimuli. For this test we derived a measure of aversion for each animal under each condition. Time spent near the three water-saturated sponges was averaged. Measure of aversion was calculated as follows: time spent near water minus time spent near irritant, divided by time spent near water, times 100. This yielded aversion measures between 0 and 100. If an animal spent equal amounts of time near water and irritant, the measure of aversion would be 0; if an animal completely avoided the irritant, the measure would be 100. We also made comparisons between average total time spent near water versus irritant for each species with each irritant, using an unpaired two-tailed t-test (Excel).

**Figure 1:**
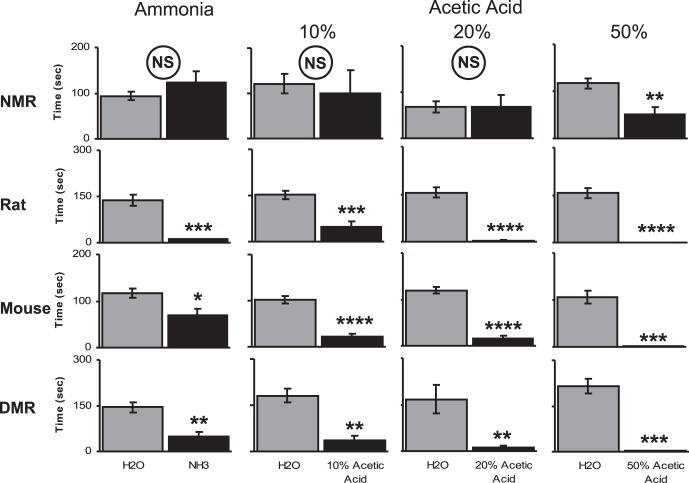
Behavioral avoidance testing with ammonia and acetic acid fumes. Animals (N = 4–10) explored a 4-arm arena that included one sponge saturated with an irritant and three sponges saturated with water. Gray bars represent the average time spent within ten centimeters of water-soaked sponges. Water data were pooled from three arms such that, for each animal there were three times as many data points for water compared to irritant. Black bars represent the average time spent within ten centimeters of the irritant-soaked sponge. Top row, naked mole-rats did not avoid fumes from ammonia, 10%, or 20% acetic acid compared to water. They did significantly avoid 50% acetic acid. Bottom three rows, rats, mice and Damaraland mole-rats showed significant avoidance to all the irritants that we tested. Error bars are standard errors of the mean. *  =  p<.05, **  =  p<.01, ***  =  p<.001, unpaired t-test.

### C Fos Immunolabeling

Animals were anesthetized with 50 mg/kg sodium pentobarbital and placed on their backs for stimulation. A sterile cotton swab was submerged in acetic acid and then placed within one centimeter to the external nares for 20 seconds. Stimulation occurred every minute for one hour. To obtain background Fos levels, control subjects were either anesthetized for one hour and then perfused, or administered a lethal dose of sodium pentobarbital and then immediately perfused. Since the animals inhaled the fumes, the entire airway was exposed to the irritants. Also, the corneas may have been stimulated to some degree.

The animals were transcardially perfused with saline followed by 4% formaldehyde in PBS. The brain and upper spinal cord was removed, post-fixed for one hour, and then stored in 30% sucrose PBS buffer at 4°C over night. The brains were mounted and frozen on a table-top microtome and serially sectioned in the transverse plane into 40 µm sections. Once cut, the tissue was stored overnight in PBS at 4°C before being processed. Serial sections were incubated in Anti-c-Fos (Ab-5) (4–17) Rabbit pAB for 48 hours (Oncogene Sciences/Calbiochem, Cambridge, MA, AB5, 25,000× in PBS with 0.25% Triton X100 and 2% normal goat serum). After incubation, tissue was rinsed in PBS and incubated in biotinylated anti-rabbit IgG for 1.5 hours and then rinsed again. The tissue was processed using the avidin-biotin complex (ABC) method for 1.5 hours using a Vectastain Elite ABC kit (Vector Laboratories, Burlingame, CA). Tissue was rinsed and stained with diaminobenzidine with nickel to obtain a gray-black stain. After processing, the tissue was mounted on subbed slides and cover-slipped. Small, ovoid-shaped objects, 5–10 μm in diameter, that were darkly stained compared to background tissue were manually counted within areas of interest using a light microscope. The person counting was blind to experimental condition. We compared diameters of labeled cells for mice and naked mole-rats in control, ammonia and 20% acetic acid conditions. Groups of twenty cells were measured for each species/condition using Image J. This data was analyzed using a two-way analysis of variance. Brain areas were defined by published stereotaxic atlases for rat [21], mouse [22], naked mole-rat [23], and published papers [24], [25]. For the caudal region of the NTS, cell counts started approximately 800 microns caudal to the area postrema (an anatomically distinct area that was not included in Fos cell counts) in the mouse and naked mole-rat. The NTS relays peripheral information in a variety of respiratory-involved sensory pathways [24]. Cell counts incorporated both lateral and medial subnuclei of the NTS. These subnuclei included the commissural and medial subnuclei, which receives information from carotid chemoreceptors [26]; and the lateral subnuclei, which are involved in the central chemoreflex pathway [25]. Further division of these subnuclei have been shown in rat and mouse [21], [22], but have not been characterized in the naked mole-rat as yet.

**Figure 2:**
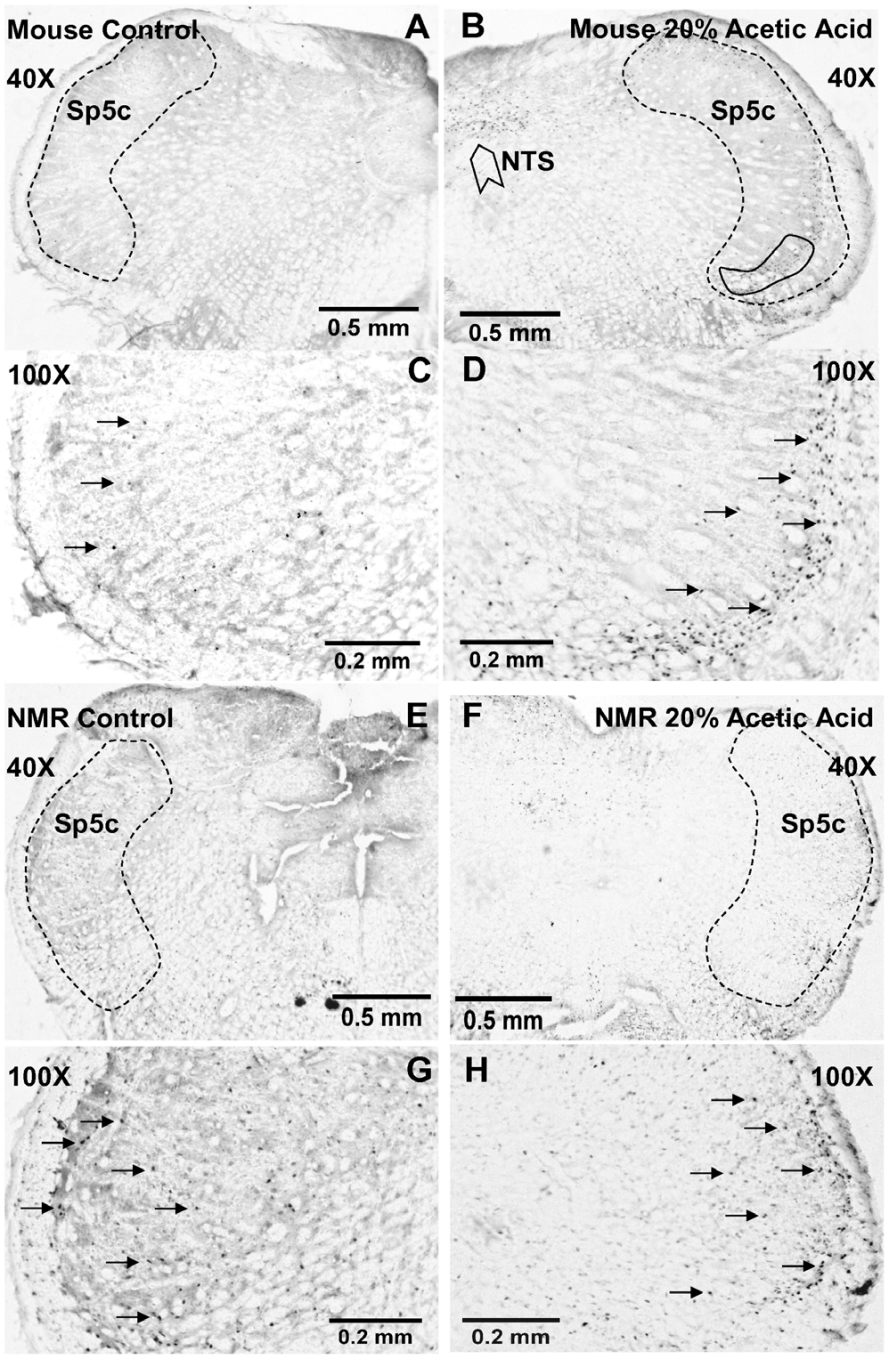
C Fos labeled sections at the level of the trigeminal pain nucleus (spinal trigeminal nucleus, caudal part, Sp5c) from mouse and naked mole-rat. A, C. Example from a control mouse at low (40X) and high (100X) magnification. Arrows in C indicate examples of C Fos positive labeled neurons. The dashed line indicates the anatomical boundary of Sp5C. Area within solid boundary indicates the estimated region of ethmoidal termination according to [29]. B,D. Example from a mouse stimulated with 20% acetic acid. Note the prominent increase of Fos labeled cells in D compared to C. In B, NTS indicates the nucleus tractus solitarius. E,G. Example from a control naked mole-rat at low and high magnification. F, H. Example from a naked mole-rat stimulated with 20% acetic acid. Note the similarity in the number of Fos labeled cells in H compared to G.

## Results

Naked mole-rats did not avoid fumes from 10% or 20% acetic acid. However, they did show significant aversion to fumes from 50% acetic acid. Rats, mice, and Damaraland mole-rats showed significant and substantial avoidance to all three acidic concentrations.

**Figure 3:**
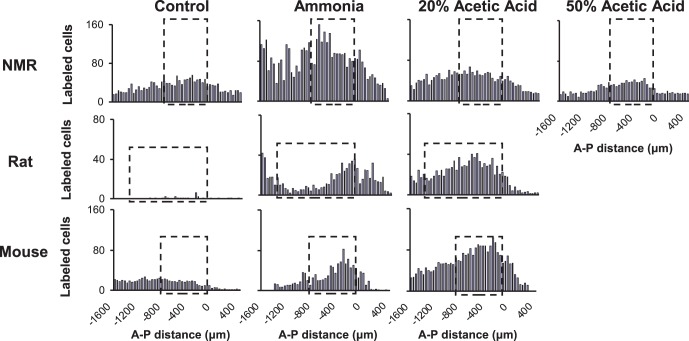
Average number of labeled cells per section through the brainstem of control and stimulated naked mole-rats, rats, and mice. The section corresponding to the rostral end of Sp5c was set as zero point and sequential sections on either side are shown in reference to zero point. A-P distance indicates anterior-posterior distance in reference to defined zero point. Dashed boxes correspond to the range of Sp5c, the area used for statistical comparisons and summarized in Figure 4. The area to the left of the dashed boxes corresponds to cervical spinal cord. The area to the right of the dashed boxes corresponds to the interpolar region of the spinal trigeminal nucleus. Note that there were obvious differences in the number of labeled cells in the control group, possibly due to differences in affinity to the antibody between species. Therefore, all analyses were made within species.

In C Fos experiments, we stimulated naked mole-rats, rats, and mice with 20% acetic acid fumes and found that rats and mice showed a significant increase in positively labeled neurons in the spinal trigeminal nucleus and the NTS. In contrast, naked mole-rats showed no significant increase for fumes from 20% acetic acid. Fumes from 50% acetic acid resulted in a significant increase in the number of labeled cells in the NTS, but, remarkably, a significant *decrease* in the trigeminal nucleus. This pattern is very different from the one associated with another irritant, ammonia. Naked mole-rats did not show a behavioral avoidance to ammonia fumes, but they did show a significant increase in C Fos labeling in the trigeminal pain nucleus.

**Figure 4:**
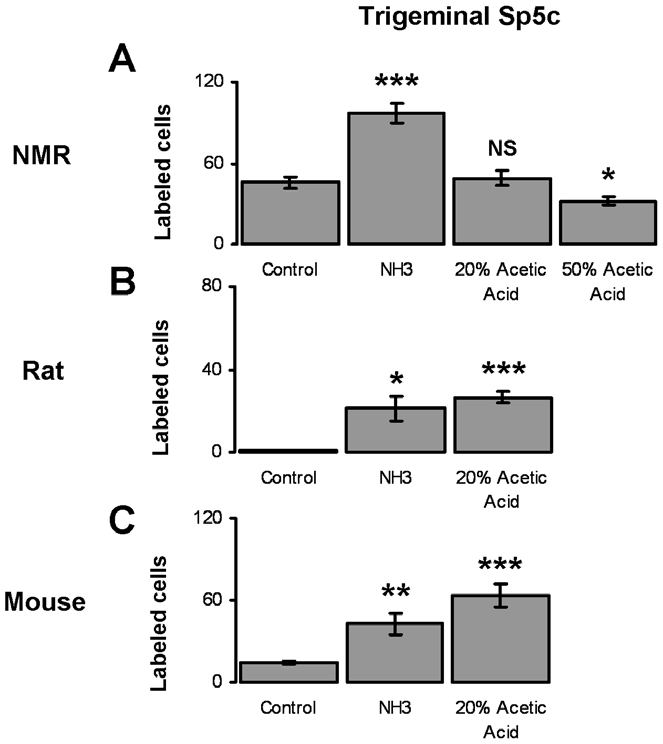
Average number of Fos labeled cells in trigeminal Sp5c in control and stimulated naked mole-rats, rats and mice. Averages correspond to the area in the dashed boxes in Figure 3. A. Naked mole-rats show a significant increase in the number of C Fos labeled neurons after stimulation with ammonia. Naked mole-rats show no increase from stimulation with 20% acetic acid and remarkably, they show a significant decrease in number of labeled cells in response to stimulation with 50% acetic acid. B and C. Rats and mice show a significant increase in the number of labeled cells in response to stimulation with ammonia and 20% acetic acid.

### Naked Mole-rats have a High Threshold for Behavioral Aversion to Acetic Acid Fumes

In a previous study, we showed that naked mole-rats did not avoid fumes from 10% ammonia, whereas laboratory rats did avoid ammonia fumes [12]. The first objective of the present study was to test responses to another relevant noxious airborne chemical irritant, acidic fumes. Acetic acid fumes, like ammonia fumes, stimulate nasal trigeminal C fibers as well as trigeminal C fibers innervating the cornea and conjunctiva [2], [27], [28]. The second objective was to expand our comparison species to include laboratory mice and another species of African mole-rat, the Damaraland mole-rat.

**Figure 5:**
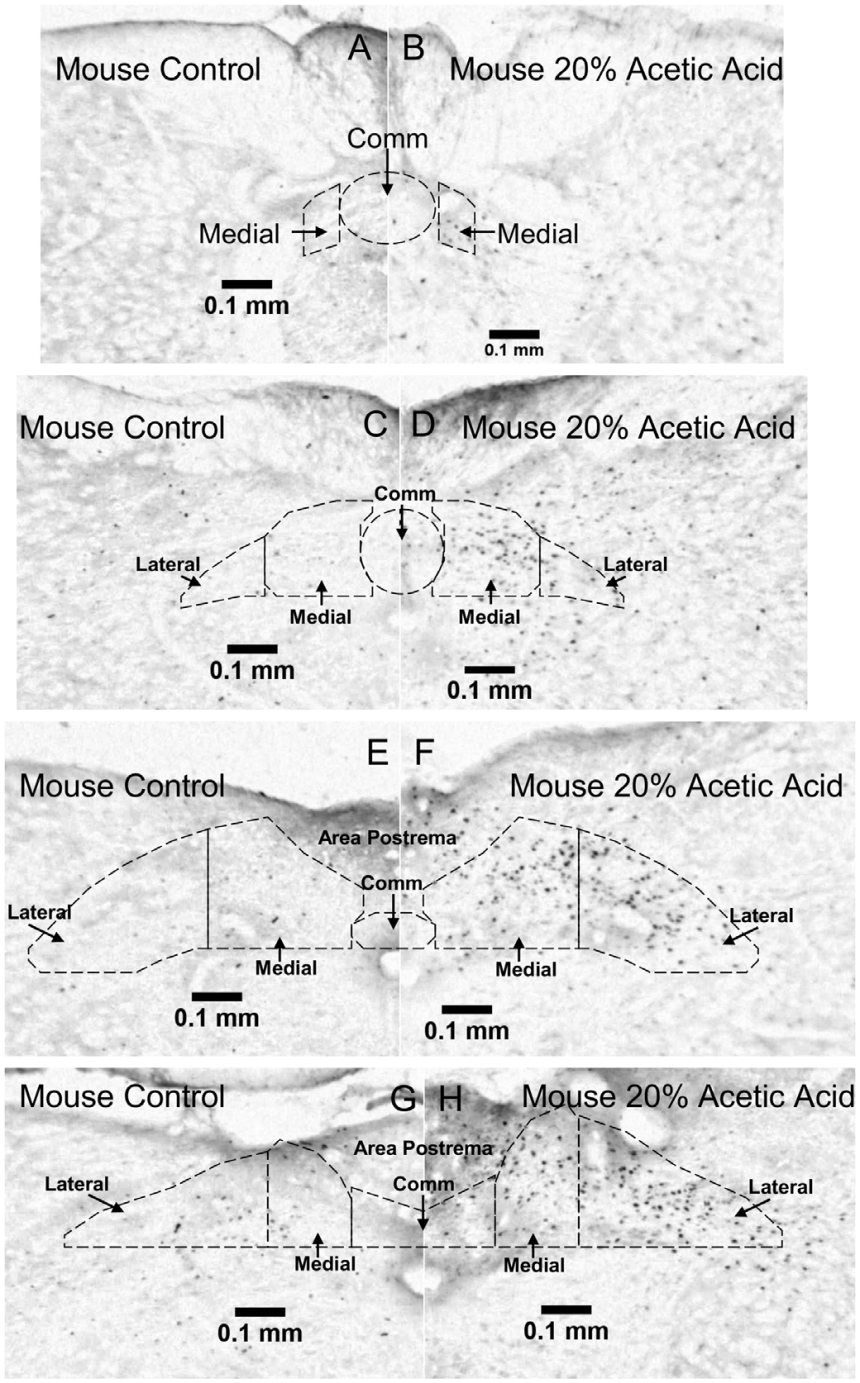
C Fos labeled sections at the level of the caudal nucleus tractus solitarius in a control and a 20% acetic acid stimulated mouse. Fos labeled sections are shown in control (A,C,E,G) versus stimulated (B,D,F,H) mouse. Sections are shown at −720 μm (A,B), −480 μm (C,D), 0 μm (E,F) and 120 μm (G,H) relative to start of the area postrema. Commissural (comm.), medial and lateral subnuclei areas are outlined according to [21], [22]. Only cells within the demarcations were counted for fos labeling. Note that our demarcations may be a bit conservative, as some labeled cells are apparent lateral to our demarcation.

**Figure 6:**
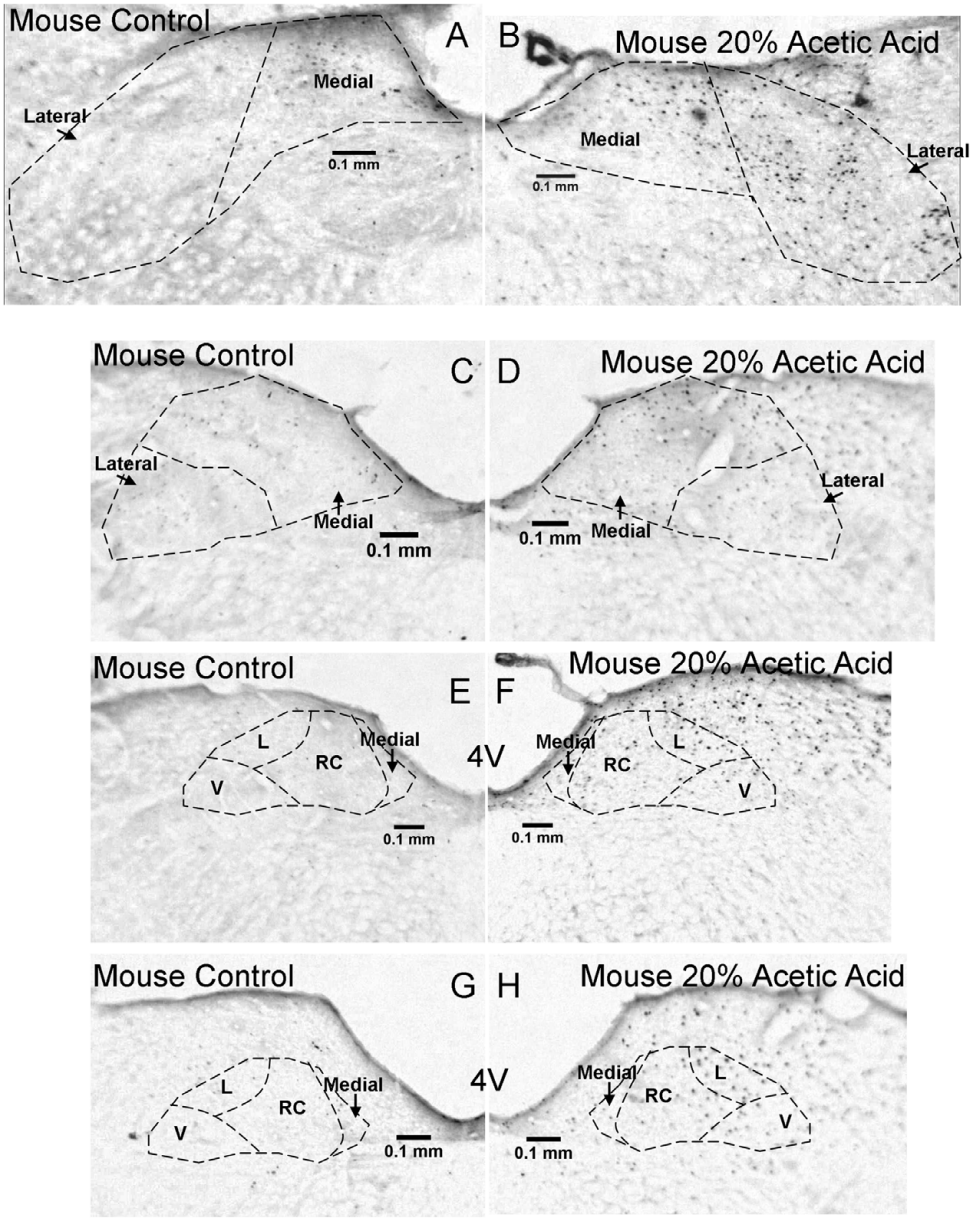
Additional c Fos labeled sections through the nucleus tractus solitarius in control and 20% acetic acid stimulated mice (continued in the rostral direction from **Figure 5**). Fos labeled sections are shown in control (A,C,E,G) versus stimulated (B,D,F,H) mouse. Sections are shown at 240 μm (A,B), 360 μm (C,D), 480 μm (E,F) and 600 μm (G,H) relative to start of the area postrema. RC  =  rostrocentral, L  =  lateral, V  =  ventral.

**Figure 7:**
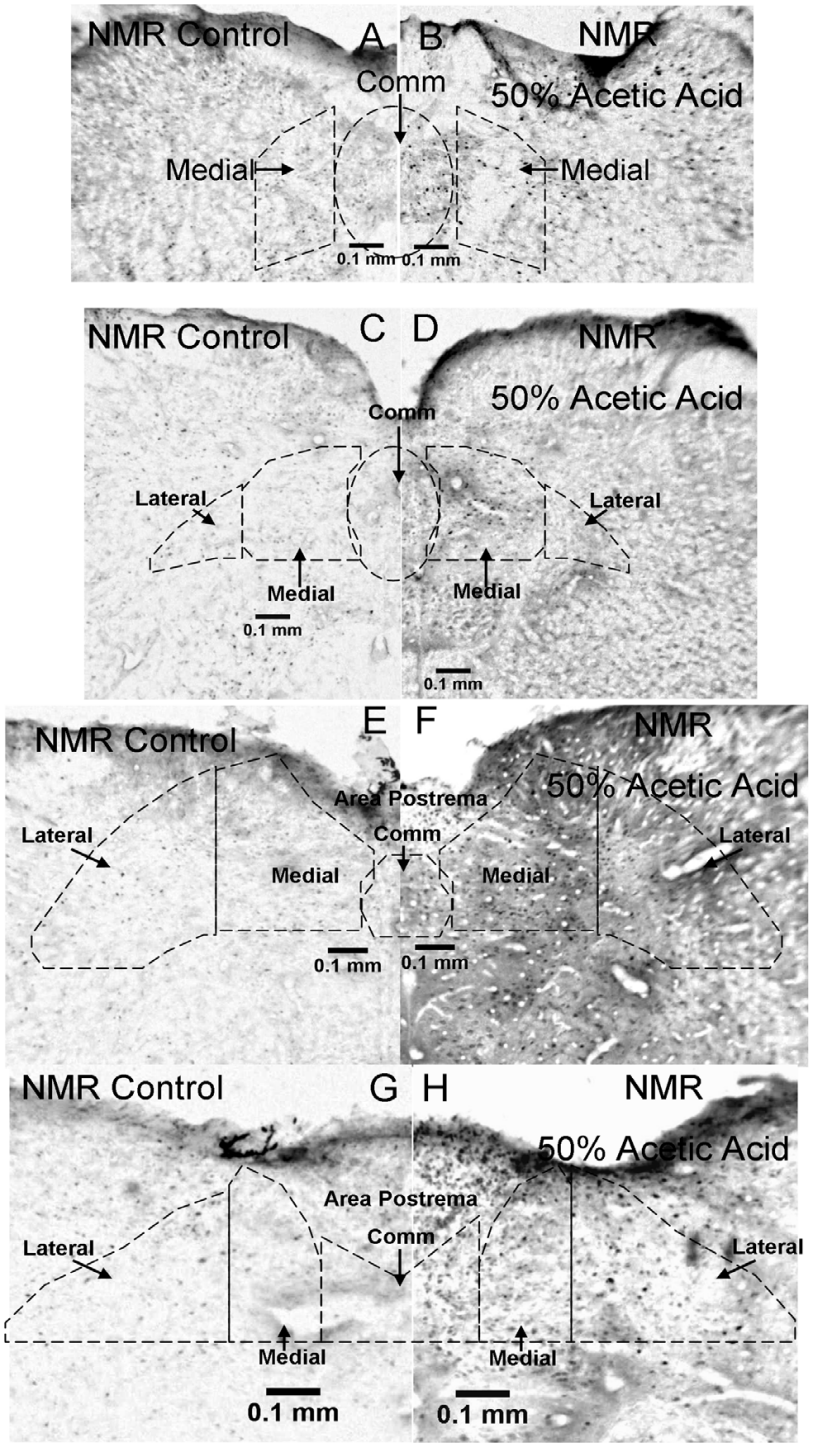
C Fos labeled sections at the level of the caudal nucleus tractus solitarius in a control and a 50% acetic acid stimulated naked mole-rat. Other aspects are the same as Figure 5 (mice).

**Figure 8:**
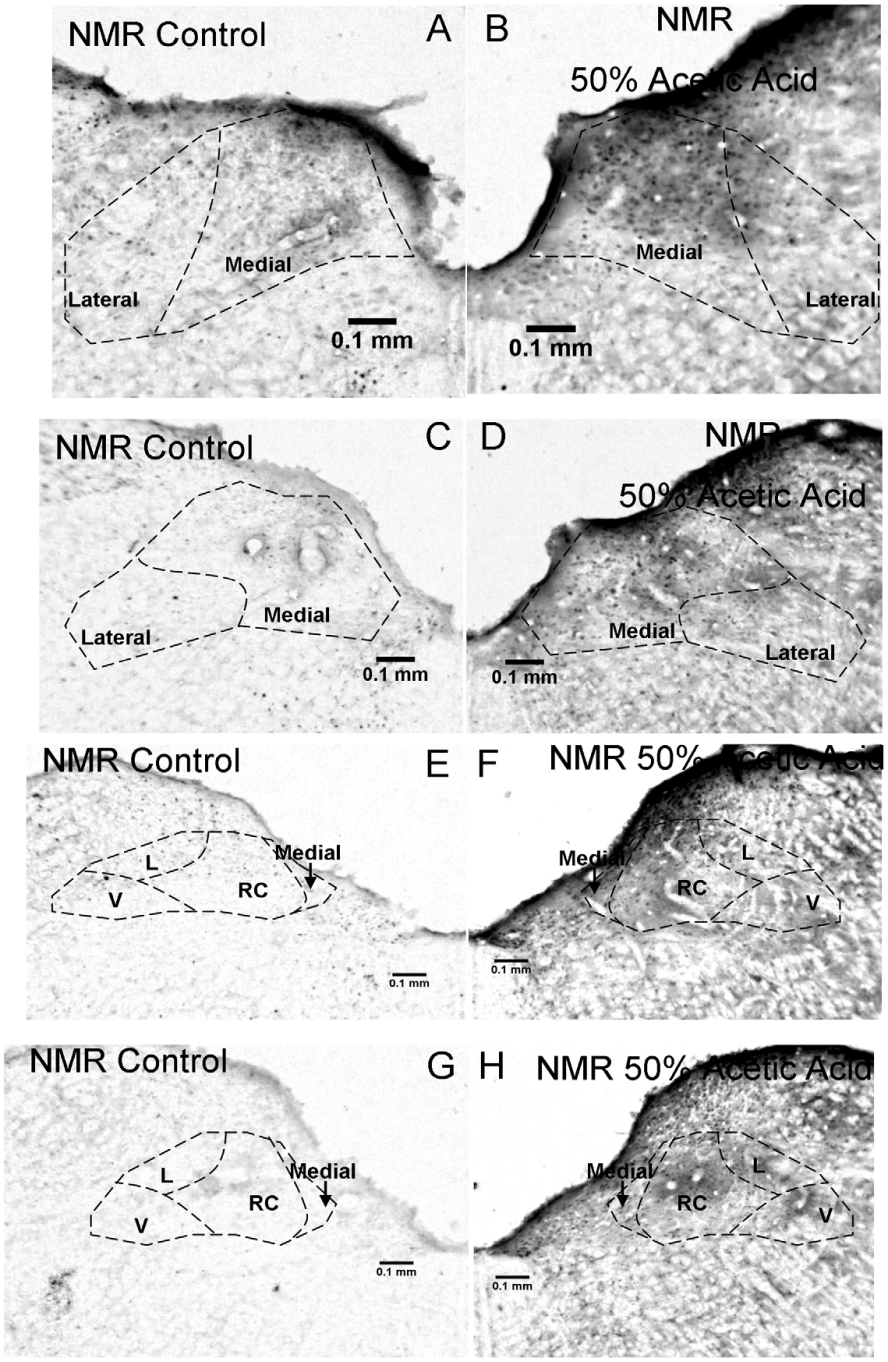
Additional c Fos labeled sections through the nucleus tractus solitarius in control and 50% acetic acid stimulated mice (continued in the rostral direction from **Figure 7**). Other aspects are the same as Figure 6 (mice).

We tested animals with fumes from ammonia and three concentrations of acetic acid, 10, 20, and 50%. A two-way analysis of variance showed a significant difference in measurements of aversion due to species **(F**_3,104_ = 6.47, p<0.001). Compared to the other species, the naked mole-rats’ aversion measures were low. There was no significant difference due to irritant type (**F**_3,104_ = 0.91, p = 0.44); nor significant interaction between species and irritant (**F**_9,104_ = 0.65, p = 0.75).

**Figure 9:**
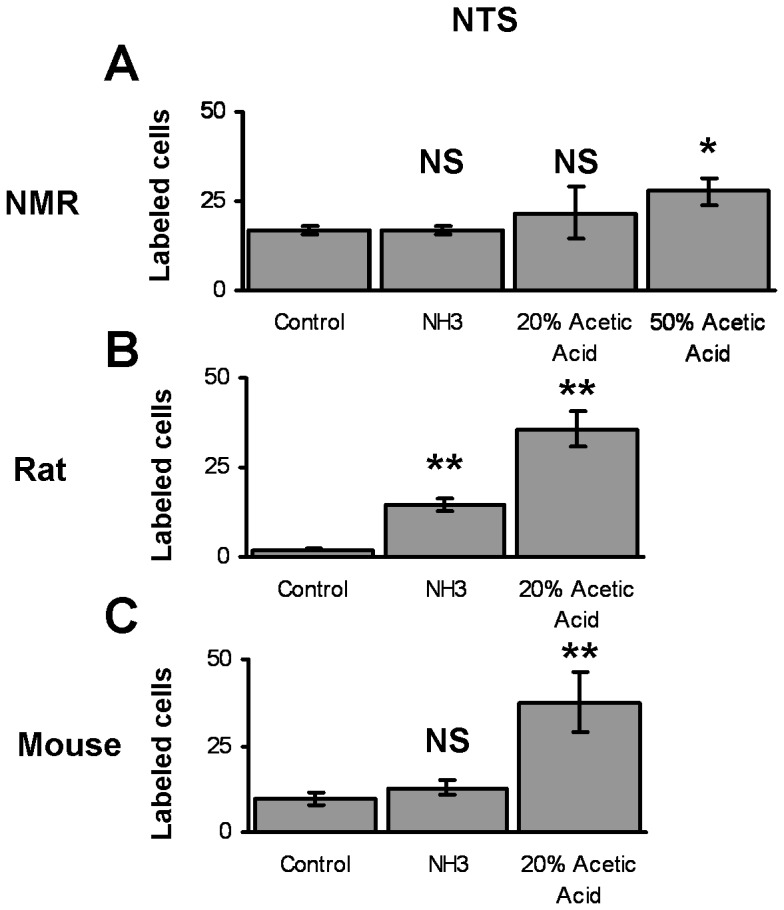
Average number of Fos labeled cells in NTS of naked mole-rats, rats, and mice. A. Naked mole-rats show no significant increase in Fos labeled cells for ammonia or 20% acetic acid. However, they do show a significant increase in NTS activity when stimulated with 50% acetic acid. This Fos data corresponds well with our behavioural data for naked mole-rats in **Figure 1**, which shows that they only avoided 50% acetic acid. B. Rats show significant increases in Fos activity in the NTS upon stimulation with ammonia and 20% acetic acid. C Mice show a significant increase in Fos labeled cells in their NTS upon stimulation with 20% acetic acid. They show no increase in activity in the NTS in response to ammonia.

The data that we used for post-hoc analyses is presented in **Figure 1**. Each bar graph displays the average amount of time spent within 10 cm of the water-soaked sponges (grey bars) versus the irritant-soaked sponge (black bars). In each case, we pooled the data from the three water sponges. Significance was determined with an unpaired t-test.

We found that the naked mole-rats only showed a significant aversion to the highest acid concentration, 50% (**Figure 1** **, top row**). For 50% acetic acid, the naked mole-rats spent an average of 117 seconds near each of the water-saturated sponges, but only 51 seconds near the 50% acetic acid-saturated sponge (**Figure 1** **, top row, far right**; t = 2.98 (df = 38), p<.01). The naked mole-rats did not avoid 10% or 20% acetic acid, or 10% ammonia. They spent the same amount of time near the sponge saturated with each of these irritants as they did with sponges saturated with water. In contrast, the rats, mice, and Damaraland mole-rats showed a significant aversion to each acid concentration and the ammonia (**Figure 1** **, bottom three rows**). Note that in **Figure 1** the ranges on the y-axis differed among species. The rats and Damaraland mole-rats spent more time near the water sponges compared to the naked mole-rats and mice. Our qualitative observation was that rats and Damaraland mole-rats moved through the arms of the maze faster than naked mole-rats and mice, thus spending less time in the arms and more time near the sponges. Also note that the group of naked mole-rats that was tested with 20% acetic acid spent less time near the water sponges compared to the time that other groups of naked mole-rats spent near the water sponges. The presence of 20% acetic acid in one sponge may have something to do with the lower amount of time spent near the water sponges. However, in our paradigm, each animal generates its own control data (water sponge versus irritant sponge). Also, the group of naked mole-rats tested with 50% acetic acid spent a similar amount of time near water sponges compared to the naked mole-rats tested with ammonia and 10% acetic acid. Hence, it is likely that the particular group of naked mole-rats tested with 20% acetic acid moved along the arms of the maze more slowly than the others.

### Naked Mole-rats Show a Decrease in Trigeminal C Fos Labeling from Stimulation with 50% Acetic Acid Fumes

We compared C Fos labeling in naked mole-rats, laboratory rats, and mice. We chose not to pursue these terminal experiments with Damaraland mole-rats because the Damaralands responded much more like rats and mice than naked mole-rats in the behavioral avoidance assay.

**Figure 2** shows cross sections through the brainstem of an example control and stimulated mouse and naked mole-rat. The stimulus was 20% acetic acid fumes. The number of positively labeled cells was substantially greater for the stimulated mouse (**Figure 2B** **, low magnification; 2D, high magnification**) compared to the control mouse (**Figure 2A,C**). The results for the mouse are consistent with previous studies that have used ammonia [12], [13] or mustard oil [14], [15] as noxious nasal stimulants. Furthermore, a main area showing increased number of labeled cells corresponds to the area associated with nasal/ethmoid innervation (e.g. [29], their **fig. 5** and 6). This area is indicated in Figure 2B by the solid boundary within Sp5c.

There were also numerous labeled cells in the nucleus tractus solitarius (NTS), which may reflect activation of pulmonary vagal nerve fibers [30]. It is also possible that cells in the NTS were stimulated by sensory fibers that innervate the oral cavity: the facial nerve, the glossopharyngeal nerve, and the lingual branch of the trigeminal nerve [31].

Sections from the control naked mole-rat (**Figure 2E,G**) had more positively labeled cells in the spinal trigeminal nucleus compared to the mouse overall (**Figure 2A,C**). However, the number of positively labeled cells was not greater for the stimulated naked mole-rat compared to the control naked mole-rat (**Figure 2G,H** **)**. Note that we found no differences in the diameters of labeled cells between mice and naked mole-rats, nor between stimulus conditions (2 way ANOVA, species: p = 0.15, stimulus: p = 0.14, species x stimulus interaction, p = 0.24).

Summary data on the number of positively labeled neurons for each species and both stimuli is shown in **Figure 3**. Each panel in **Figure 3** shows average cell counts for each 40 µm section through the brainstem from cervical spinal cord (left) to the interpolar region of the trigeminal nucleus (right). The dashed boxes indicate the trigeminal pain nucleus (spinal trigeminal nucleus, caudal part, Sp5c). For each species, a greater number of labeled cells can be observed in the Sp5c for the ammonia group compared to the control group. For stimulation with 20% acetic acid, only the rats and mice showed a substantial increase compared to their respective control groups while the naked mole-rats remained near control values. An additional group of naked mole-rats was tested with 50% acetic acid, and that group showed a reduction in the number of labeled neurons compared to the control group.

The average numbers of Fos labeled neurons across all trigeminal Sp5c slices are shown in **Figure 4**. For naked mole-rats (**Figure 4A** **)**, there are significantly more positively labels cells in the ammonia group compared to the control group (on average, 96.6 vs 45.8, t = −6.28, df = 8, p<.001). Importantly, there is no significant difference between the average number of labeled neurons for the naked mole-rats stimulated with 20% acetic acid compared to those in the control group (48.9 vs 45.8, t = −0.44, df = 8, NS).

The Fos data from stimulating naked mole-rats with acidic fumes appears to be inconsistent with the Fos data from ammonia and the behavioral avoidance results: Behaviorally, naked mole-rats respond in the same way to both the ammonia and the 20% acetic acid in that they do not avoid either. Yet, the group stimulated with ammonia had significantly more labeled neurons than control, while the group stimulated with 20% acetic acid did not.

The results from naked mole-rats that were stimulated with 50% acetic acid not only failed to show an increase in Fos labeling, they showed a significant decrease compared to the control group (**Figure 4A**). The average number of labeled neurons for the 50% acetic acid group was 31.8 compared to 45.8 for control (t = −6.28, df = 10, p<.05). Hence, the only irritant stimulus that we tested that evoked avoidance in naked mole-rats actually decreased labeling in the trigeminal pain nucleus.

The summary data for laboratory rats and mice is more straightforward (**Figure 4B,C**). Both rats and mice showed significantly more labeled neurons in their respective ammonia and acetic acid groups, compared to their control groups.

### Naked Mole-rats Show an Increase in C Fos Labeling from Stimulation with 50% Acetic Acid Fumes in the Nucleus Tractus Solitarius

Examples of Fos labeling through the caudal region of the NTS are shown for mice in **Figure 5**. Sections through the rostral part of the NTS for mice are shown in **Figure 6**. For naked mole-rats, sections through the caudal NTS are shown in **Figure 7**, and sections through the rostral NTS are shown in **Figure 8**.

The average numbers of labeled neurons in the caudal NTS for naked mole-rats, rats, and mice are shown in **Figure 9**. For the naked mole-rats (**Figure 9A**), 50% acetic acid was the only stimulant tested that resulted in a significant increase in labeled neurons compared to control (27.6 vs 16.8, t = −2.96, df = 9, p<.05). The number of labeled neurons in naked mole-rats tested with ammonia and 20% acetic acid were not significantly different from the number of labeled neurons in control naked mole-rats. Hence, fumes from 50% acetic acid were able to increase the number of Fos labeled cells in the NTS, which was surprising to us given the mutation in naked mole-rat Nav1.7.

In contrast to the naked mole-rats, laboratory rats showed significant increases in the number of labeled neurons for both ammonia and 20% acetic acid (**Figure 9B**). Laboratory mice showed a significant increase in NTS activity in response to 20% acetic acid, but not in response to ammonia stimulation (**Figure 9C** **)**. Interestingly, mice showed a higher behavioural tolerance to ammonia fumes than acetic acid fumes, spending relatively more time near the ammonia sponge (**Figure 1**). Previous studies have shown that, in the laboratory, mouse cages accumulate much higher concentrations of ammonia than rat cages [32], [33]. Hence, it seems possible that mice in the wild live under chronically high levels of ammonia in their nests which might result in a higher tolerance to ammonia behaviourally and physiologically.

## Discussion

Naked mole-rats display a number of putative adaptations for living under chronic, low O_2_/high CO_2_ conditions in their crowded burrows. For low O_2_, these include a low resting metabolic rate [34], hemoglobin with a high affinity for O_2_ [35], and hypoxia tolerant brain tissue [36]–[38]. With regards to living under high CO_2_/acidosis, putative adaptations include voltage-gated sodium channels that inhibit spiking in pain fibers under acidic conditions [4] and a lack of neuropeptides in pain fibers [3], [39] which would be released during acidosis in other species, generating a painful burning sensation [40], [41].

The present study focused on behavioral, trigeminal, and NTS responses to acidosis from exposure to acetic acid fumes in naked mole-rats. The main findings were: 1) naked mole-rats showed behavioral aversion to acidic fumes, but only at a much higher level compared to rats and mice; 2) aversion in the naked mole-rats appears to have been driven not via activation of the trigeminal brainstem pain pathway as in other mammals; and 3) the patterns of behavioral aversion and trigeminal Fos labeling were reversed for naked mole-rats tested with 50% acetic acid compared to naked mole-rats tested with ammonia. Exposure to 50% acetic acid fumes generated aversion and a decrease in Fos labeling in the trigeminal pain nucleus, whereas ammonia generated no aversion but an increase in Fos labeling in the trigeminal nucleus. This is intriguing considering that in the mammalian nasal epithelium, c fiber nociceptors that signal pain/irritation usually respond to both acid and ammonia (and capsaicin) [10], [11], [42], [43]. Both acid and ammonia activate TRPV1 and TRPA1 receptors on c fiber nociceptors [2], [44]–[47]. Arai, et al. [47] showed that TRPV1 underlies responses to acids in the larynx. It is possible that different types of acids activate different types of receptors but there are multiple acid sensors and as of yet, the literature does not conclusively support this idea (For review, see [48]).

The naked mole-rat trigeminal pain pathway has several anomalies that can account for this species’ apparently inconsistent behavioral and c Fos responses to acetic acid and ammonia. The most recently identified anomaly is a gene variant in the naked mole-rat voltage-gated sodium channel, Nav1.7 which causes inhibition of spike initiation under acidic conditions [4]. This finding is consistent with a previous report that low pH saline failed to drive spikes in cutaneous c fibers, whereas capsaicin triggered normal spiking in these fibers [3]. Acid insensitivity at the level of the afferents might also account for the Fos results from acidic stimulation of the trigeminal nerve in the present study. Fumes from 20% acetic acid did not increase post-synaptic, activity-driven Fos labeling in the trigeminal nucleus of naked mole-rats. Remarkably, the significant decrease in labeled trigeminal cells from 50% acetic acid suggests that acidification can even suppress baseline activity.

The gene variant in naked mole-rat Nav1.7 can account for high behavioral thresholds and low c Fos labeling in the trigeminal nucleus for acidic stimuli. Under low pH, naked mole-rat Nav1.7 is blocked by protons [4] preventing action potential conduction. However, under physiological pH, the Nav1.7 channels respond normally. Thus, sensory cells are able to initiate and conduct action potentials in response to stimulation by non-acidic irritants such as capsaicin [3] and ammonia. However, another striking anomaly has been identified in the naked mole-rat pain pathway that can have a role in responses to these irritants. C fibers in the trigeminal (and dorsal root) ganglia of naked mole-rats naturally lack certain neuropeptides associated with pain/irritant signaling [39]. These neuropeptides include Substance P and Calcitonin Gene-Related Peptide, and their selective lack appears to involve specific deletions in gene promoters [49]. However, c fibers in naked mole-rats retain glutamate [50], which is able to trigger activity in post-synaptic cells in the dorsal horn [3] and trigeminal nucleus [12]. Hence, irritants such as ammonia and capsaicin can trigger spikes in c fiber afferents and activity in post-synaptic targets which can be detected via Fos labeling (present study) or patch clamp recording [3].

Even though irritants other than acid can activate c fibers and post-synaptic targets in naked mole-rats, without the neuropeptides, pain and aversive behaviors are extremely reduced or lacking. Thus, injecting the foot skin with capsaicin [3], or stimulating the nasal epithelium with ammonia fumes results in virtually no behavioral responses [12] (and present study). We previously showed the important role for one of the lacking neuropeptides, Substance P, in c fiber-mediated pain behavior. Introducing Substance P into the spinal cord of naked mole-rats by gene therapy or by intrathecal injection resulted in rescue of pain behaviors in response to capsaicin application [3], [50], as well as rescue of itch behaviors to application of histamine [51]. These procedures had no effect on acid insensitivity, which is consistent with a different mechanism (Nav1.7) for acid insensitivity. Hence, we suggest that two distinct mechanisms account for behavioral insensitivity to acid and ammonia/capsaicin: proton block of Nav1.7 for acid, and lack of neuropeptides for ammonia/capsaicin. In regard to c fos labeling, it is very intriguing that naked mole-rats show an increase in c fos labeling from stimulation with ammonia even though they show no behavioral avoidance to ammonia. Our current hypothesis on this apparent incongruity is that glutamate release from c fibers responding to ammonia is sufficient to drive fos production in the spinal trigeminal nucleus. However, aversive behaviors from ammonia stimulation appear to require the neuropeptides that are congenitally absent from the trigeminal c fibers in naked mole-rats. Interestingly, naked mole-rats do show behavioral aversion to nicotine [12], a potent trigeminal irritant stimulus associated with A delta fibers, not peptidergic c fibers [52], [53].

An interesting evolutionary question is, why do naked mole-rats have what appear to be redundant mechanisms for stifling c fiber-mediated pain? Our working hypothesis is that the anomalies in the naked mole-rat pain pathway are adaptations for living in a chronically high CO_2_/acidic environment. Under this hypothesis, insensitivity to ammonia, and for that matter capsaicin, may be an epiphenomenon since many of the same fibers respond to CO_2_, ammonia, and capsaicin. It would seem that either the lack of neuropeptides, or the altered Nav1.7 would achieve acid insensitivity, so why both?

The result that naked mole-rats do avoid fumes from 50% acetic acid is interesting. The results from Fos labeling in the NTS suggest that this pathway may be involved in mediating this species’ aversion behavior based on the correlation between labeling and behavior. In naked mole-rats, 50% acetic acid drives both behavioral aversion and a significant increase in the number of Fos labeled neurons in the NTS. For a lower concentration of acetic acid (20%), there was no aversion and no increase in labeling. Alternatively, an increase in Fos labeling in the NTS may be related to pH effects on respiration centers [54], [55] or oral afferents [31].

Acid-driven activity in the NTS of the naked mole-rat is interesting given the gene variant in naked mole-rat Nav1.7. One might have expected that acidity would be unable to drive action potentials in pulmonary or oral afferent nerves, similar to the inability to drive action potentials in nasal trigeminal nerves. The difference in how sensory fibers associated with the trigeminal nucleus and the NTS respond to acidification may result from differences in distributions and/or densities of Nav1.7 and other sodium channels (Nav1.8, Nav1.9).

Another interesting possibility for avoidance of high concentrations of acetic acid by naked mole-rats is avoidance due to stimulation of odor or taste receptors. We previously showed that naked mole-rats have comparable olfactory discrimination abilities to rats for several odorant pairs, including ammonia versus water [12]. To the best of our knowledge, no one has examined taste abilities in the naked mole-rat.

In conclusion, naked mole-rats have a high threshold for behavioral avoidance to acidic fumes and an apparent lack of acid-driven activity in the trigeminal pain nucleus. However, activity in the NTS is consistent with behavioral avoidance. The high threshold for airborne acidic fumes is consistent with an adaptation for living under chronically acidic conditions.
